# Transcription Factor VviMYB86 Oppositely Regulates Proanthocyanidin and Anthocyanin Biosynthesis in Grape Berries

**DOI:** 10.3389/fpls.2020.613677

**Published:** 2021-01-13

**Authors:** Jing Cheng, Keji Yu, Ying Shi, Jun Wang, Changqing Duan

**Affiliations:** ^1^Center for Viticulture and Enology, College of Food Science and Nutritional Engineering, China Agricultural University, Beijing, China; ^2^Key Laboratory of Viticulture and Enology, Ministry of Agriculture and Rural Affairs, Beijing, China; ^3^Beijing Advanced Innovation Center for Tree Breeding by Molecular Design, Beijing Forestry University, Beijing, China

**Keywords:** flavonoid, grape (*Vitis vinifera* L.), leucoanthocyanidin reductase, light, regulation, R2R3-MYB

## Abstract

Proanthocyanidins (PAs) and anthocyanins are two vital groups of flavonoid compounds for grape berries and red wines. Several transcription factors (TFs) have been identified to be involved in regulating PA and anthocyanin biosynthesis in grape berries. However, research on TFs with different regulatory mechanisms for these two biosynthesis branches in grapes remains limited. In this study, we identified an R2R3-MYB TF, VviMYB86, whose spatiotemporal gene expression pattern in grape berries coincided well with PA accumulation but contrasted with anthocyanin synthesis. Both *in vivo* and *in vitro* experiments verified that VviMYB86 positively regulated PA biosynthesis, primarily by upregulating the expression of the two leucoanthocyanidin reductase (LAR) genes in the Arabidopsis protoplast system, as well as in *VviMYB86*-overexpressing grape callus cultured under 24 h of darkness. Moreover, VviMYB86 was observed to repress the anthocyanin biosynthesis branch in grapes by downregulating the transcript levels of *VviANS* and *VviUFGT*. Overall, VviMYB86 is indicated to have a broad effect on flavonoid synthesis in grape berries. The results of this study will help elucidate the regulatory mechanism governing the expression of the two LAR genes in grape berries and provide new insights into the regulation of PA and anthocyanin biosynthesis in grape berries.

## Introduction

Flavonoids are an important group of secondary metabolites in plants; they include proanthocyanidins (PAs, also known as condensed tannins), anthocyanins, and flavonols, that largely accumulate in the berries, seeds, leaves, flowers, and bark of many plant species (Dixon et al., 2005). Grape (*Vitis vinifera* L.) is one of the most widely cultivated fruits worldwide and has important economic value. For grape berries and red wines, PAs and anthocyanins strongly contribute to the astringent taste and red coloration, respectively (Peters and Constabel, 2002; Vidal et al., 2003). Furthermore, both PAs and anthocyanins confer potential beneficial effects on human health due to their antioxidant capacities and radical scavenging functions (Cos et al., 2004; Butelli et al., 2008). Therefore, the biosynthesis and regulation of PAs and anthocyanins in grape berries have attracted considerable amounts of attention (Cavallini et al., 2015; Wei et al., 2020).

PAs, as well as their building blocks and anthocyanins, are synthesized *via* the flavonoid metabolic pathway, and they share most of the same steps (Supplementary Figure 1; Dixon et al., 2005; Li et al., 2016). Leucoanthocyanidins, which are the first branch point between the PA and anthocyanin biosynthesis pathways, can be converted into 2,3-*trans*-flavan-3-ols [such as (+)-catechin] by leucoanthocyanidin reductase (LAR) or can be oxidized to generate extremely unstable anthocyanidins by anthocyanidin synthase (ANS). Next, anthocyanidins are utilized to produce 2,3-*cis*-flavan-3-ols [such as (−)-epicatechin] by anthocyanidin reductase (ANR) or are employed by UDP-glucose: flavonoid-3-*O*-glucosyltransferase (UFGT) to generate stable anthocyanins (Xie et al., 2003).

LAR and ANR are two key enzymes involved in PA biosynthesis (Tanner et al., 2003; Xie et al., 2003). In grapes, ANR is primarily responsible for the total amount of PAs, and is encoded by the gene *VviANR*, which is expressed throughout early flower and berry development (Xie et al., 2003; Bogs et al., 2005). Unlike Arabidopsis, which lacks LAR in PA biosynthesis, grape possesses two LAR enzymes (VviLAR1 and VviLAR2) with similar functions—not only producing 2,3-*trans*-flavan-3-ols from leucoanthocyanidins but also controlling the degree of PA polymerization (Yu et al., 2019). However, the expression of the genes encoding VviLAR1 and VviLAR2 is modulated in a temporal- and tissue-specific manner in grape berries (Bogs et al., 2005). In grape berry skins and seeds, *VviLAR2* is highly expressed during the second half of the PA biosynthesis phase, when *VviLAR1* is at a low transcript level (Bogs et al., 2005). These results imply that these three PA branch-specific genes might be differentially regulated in grape berries.

Several major PA and anthocyanin biosynthesis genes, such as *ANS*, *LAR*, *ANR*, and *UFGT*, are strictly regulated at the transcription level, and one of the regulatory mechanisms involves transcription factors (TFs) that regulate the expression of corresponding structural genes (Xu et al., 2014). To date, multiple TFs belonging to the MYB, bHLH, WD, MADS, and WRKY families have been characterized to regulate PA and anthocyanin biosynthesis (Wei et al., 2020). Among these TFs, those from the MYB family have been studied most extensively. Several MYB TFs have been demonstrated to be positively involved in PA and/or anthocyanin synthesis in grape berries. PA synthesis is regulated specifically by VviMYBPA1, VviMYBPA2, and VviMYBPAR, which have been isolated through homologous cloning based on Arabidopsis PA-related genes (Bogs et al., 2007; Terrier et al., 2009; Koyama et al., 2014). In addition, target genes of the three MYB TFs in grape berries are *VviLAR1* and *VviANR*, although the expression pattern of *VviMYBPAR* is more similar to that of *VviLAR2* throughout berry development (Bogs et al., 2007; Terrier et al., 2009; Koyama et al., 2014). These results indicate that additional putative regulators that participate in PA synthesis may exist, primarily by controlling the expression of *VviLAR2*. With respect to anthocyanins, the TF VviMYBA1 directly controls the expression of *VviUFGT* in grape berries (Cutanda-Perez et al., 2009). The abovementioned MYB TFs all possess a single regulatory function in flavonoid biosynthesis of grapes—regulate either PA synthesis or anthocyanin synthesis. In contrast with these functionally specialized MYB TFs, some MYB TFs have a broad effect on flavonoid synthesis *in planta*. For instance, *Populus tomentosa* PtMYB86, *Tartary buckwheat* FtMYB15, and *Freesia hybrida* FhMYB5 can promote both anthocyanin and PA branches at the same time (Luo et al., 2018; Li et al., 2019; Wang et al., 2019). In grape berries, VviMYB5a and VviMYB5b are involved in both anthocyanin and PA synthesis, and their regulation of anthocyanins and PAs is positive (Deluc et al., 2006, 2008).

In addition to MYB activators, several R2R3-MYB TFs have been identified as negative regulators of the flavonoid pathway. Generally, R2R3-MYB repressors can be divided into two clades: AtMYB4-like and FaMYB1-like (Chen et al., 2019). The two clades of R2R3-MYB repressors show variations in their conserved element termed the A2 box or element 3, which is located in the R3 domain (Cavallini et al., 2015). In the AtMYB4-like clade, the sequence of this element is DNEI, whereas the sequence is DNEV in the FaMYB1-like clade (Chen et al., 2019). In addition to the difference in amino acid in the conserved element, these two R2R3-MYB-type repressors are also different in their functions. AtMYB4-type R2R3-MYB repressors negatively affect the gene expressions of lignin and general phenylpropanoid pathway, whereas FaMYB1-type R2R3-MYB repressors downregulate the gene expressions of anthocyanin and/or PA biosynthetic pathway (Cavallini et al., 2015; Jun et al., 2015; Zhou et al., 2019). Unlike most flavonoid R2R3-MYB activators, R2R3-MYB repressors seem to repress more than one end product. Furthermore, MYB TFs with opposite effects on different biosynthesis branches of flavonoids have been observed in other plant species recently, such as *Narcissus tazetta* NtMYB3, *Medicago truncatula* MtPAR, and *Camellia sinensis* CsMYB5a. All of these TFs promote PA biosynthesis but repress other flavonoid biosynthesis branches, such as flavonols, isoflavones, and anthocyanins (Li et al., 2016; Jiang et al., 2018; Anwar et al., 2019). In grape berries, TFs that have opposite regulatory mechanisms to PA and anthocyanin pathways are being identified.

Genetic factors and several abiotic factors such as light, temperature, and water can also influence PA and anthocyanin biosynthesis *in planta* (Downey et al., 2006). Among these environmental stimuli, light is the major factor associated with flavonoid accumulation and the composition of grapes. The results of several field experiments have confirmed that exposure of grape bunches to light can enhance the expression levels of corresponding structural and regulatory genes involved in the PA and anthocyanin biosynthesis pathways, as well as promote PA and anthocyanin accumulation in berries (Fujita et al., 2007; Matus et al., 2009; Lee, 2017). Recently, our research group investigated the effects of different light conditions on phenolic metabolism in grape berries by applying integrated transcriptomics and pathway-level metabolomics (Sun et al., 2017, 2019). The results of these studies demonstrate that the transcript abundances of *VviLAR1* and *VviANR* have a significant positive correlation with the altered accumulation of PAs in light exclusion-treated grape berries, whereas the expression of *VviLAR2* is not sensitive to changes in light (Additional ‘file’ 8: Supplementary Table 6 in Sun et al., 2019). Moreover, to systematically investigate additional putative regulators that may regulate flavonoid biosynthesis in grape berries, a genome-wide co-expression analysis between flavonoid pathway-related synthesis genes and regulatory genes was constructed; notably, TFs belonging to MYB, MYC, WRKY, MADS, and HD-Zip were the most abundant significantly co-expressed TFs (Sun et al., 2017, 2019).

In this study, using both *in vivo* and *in vitro* experiments, we investigated the role of VviMYB86 in the regulation of flavonoid biosynthesis in grape berries. We demonstrated that *VviMYB86* regulated PA and anthocyanin biosynthesis oppositely in grape berries; this TF significantly increased PA contents, primarily by enhancing the expression levels of the two LAR genes, whereas it reduced anthocyanin contents by downregulating the expression of *VviANS* and *VviUFGT*. Taken together, our findings provide insights into the regulatory mechanisms mediating anthocyanin and PA biosynthesis related to R2R3-MYB TFs.

## Materials and Methods

### Plant Materials and Growth Conditions

Grape materials of *V. vinifera* L. cv. Cabernet Sauvignon (CS) were collected from the experimental vineyard at the Shangzhuang Experimental Station of China Agricultural University in Haidian district, Beijing (40°14′ N, 116°20′ E, altitude 49 m), during the 2019 growing season. The own-rooted CS vines were planted in 2011 and were spaced at 2.5 m × 1.2 m, arranged in north-south rows. The training system and irrigation method applied in the vineyard were a modified vertical shoot positioning system and drip irrigation, respectively. Pest and nutrition management in the vineyard was undertaken in keeping with the local industry standard. Grapevine organs, including flowers, tendrils, stems, young leaves (10-day aged) and mature leaves (30-day aged) and berries were collected at E-L 23 (full blooming with 50% cap off) from at least nine randomly selected grapevines at distinct developmental time points. The roots were collected at E-L 23 from the underground parts of another nine randomly selected grapevines in the same vineyard. Berries were sampled at six developmental stages, including E-L 29 (peppercorn size), E-L 31 (pea-size), E-L 34, E-L 35 (early version), E-L 37 (end of version) and E-L 38 (commercial maturity), which corresponded to 5, 15, 43, 63, 80, and 98 days after anthesis, respectively. The sampling time was fixed at 8:00 to 9:00 am. After sampling, the grapevine organs and berry samples were placed into an ice box and transported into the laboratory within 2 h (Wen et al., 2015). After the samples arrived at the laboratory, they were immediately frozen in liquid nitrogen and stored at −80°C for further analysis. For the berries corresponding to E-L 31 to E-L 38, skin and seed samples were obtained by peeling fresh flesh, and the dissected skins and seeds were immediately immersed in liquid nitrogen before being stored at −80°C. Three biological replicates were taken for each sample.

The grape callus used in the present study was induced from berry skins of CS following the method described by Wang et al. (2015). The subculture medium comprised solid B5 medium supplemented with 30 g/L sucrose, 2.5 g/L acid-hydrolyzed casein, 3.0 g/L phytagel, 0.1 mg/L α-naphthylacetic acid, and 0.2 mg/L kinetin, and the pH was 5.9–6.0. The culture conditions were 24-h dark at a temperature of 24°C, and the callus was subcultured every 25 days.

Wild-type *Arabidopsis thaliana* (ecotype Col-0) was grown in soil in a growth chamber under cool-white fluorescent lamps providing a 16/8-h light/dark photoperiod at 25 ± 1°C.

### Cloning and Bioinformatic Analysis of VviMYB86

On the basis of the predicted cDNA sequence of *VviMYB86* (GenBank accession No. XM_002280991) in the NCBI reference sequence database and the genomic sequence of CS (VIT_207s0005g02480)^1^, the open-reading frame (ORF) of *VviMYB86* was cloned *via* gene-specific primers (VviMYB86-F/R) from a CS cDNA library previously constructed by our lab (Mu et al., 2014). The resulting amplicons were cloned into a pMD19-T vector (Tsingke, China) for sequencing. The primers employed in the present study are listed in Supplementary Table 1.

Phylogenetic analysis was conducted using the neighbor-joining method in MEGA version 7.0 (Kumar et al., 2016). The conserved domains of the VviMYB86 protein were scanned by the Inter-ProScan program^2^. The theoretical molecular weight and isoelectronic point were calculated using the ProtParam tool^3^. For multiple sequence alignment analysis, the amino acid sequence of VviMYB86 and those of other MYB homologs from different plant species retrieved from the NCBI database were aligned using DNAMAN 6.0 software (Lynnon Corporation, United States).

### Isolation and Bioinformatic Analysis of the VviMYB86 Promoter (pVviMYB86) Region

Grape genomic DNA was extracted from CS berries using a New Rapid Plant DNA Extraction Kit (BioTeke, China) and then used as template for cloning. The specific primers (Supplementary Table 1) were designed for amplification based on the predicted sequence from the NCBI reference sequence (NC_012013.3) and genomic sequence of CS^4^. The PCR products that were approximately 1,300 bp from the start codon, which were obtained using the Pfu DNA polymerase (Tiangen, China), were inserted into the pMD19-T vector (Tsingke, China) for sequencing validation. The *cis*-acting elements on p*VviMYB86* were predicted by the PlantCARE website^5^. Sequence data from this article have been deposited in GenBank under accession number MW046258.

### Total RNA Extraction, Reverse Transcription, and Quantitative Real Time PCR (qRT-PCR) Analysis of Gene Expression

All frozen plant samples (i.e., grapevine organs, berry skins, berry seeds, and grape calluses) were ground in liquid nitrogen, and total RNA was subsequently extracted by using a Universal Plant Total RNA Extraction Kit (BioTeke, China). The quality and concentration of the obtained RNA were detected using agarose gel electrophoresis and a NanoDrop 2000 spectrophotometer (Thermo Fisher, United States), respectively. The RNA employed for further analysis appeared as clear and bright bands in the agarose gel, the value of OD260/OD230 was more than 1.8, and the value of OD260/OD280 was between 1.8 and 2.1 (Meng et al., 2020). A total amount of 1 μg RNA was utilized for reverse transcription reaction using HiScript^®^ IIQ RT SuperMix for qPCR (+ gDNA wiper) (Vazyme, China). qRT-PCR was performed using ChamQ Universal SYBR qPCR Master Mix (Vazyme, China) and an Applied Biosystems 7300 Real Time PCR System (Thermo Fisher, United States). Each qRT-PCR reaction (20 μL) consisted of 10 μL ChamQ Universal SYBR qPCR Master Mix, 2 μL of cDNA, 7.2 μL of ddH_2_O, and 0.8 μL of a primer mixture (equal amounts of forward primer and reverse primer, 10 mM). The specific PCR procedure was performed as described by Sun et al. (2015). Each RNA sample was subjected to three independent reactions in qRT-PCR analysis. The analysis followed the method described in a previous report (Ruijter et al., 2009). *VviUbiquitin1* was employed as the reference gene (Bogs et al., 2005). The gene-specific primers used in this study are listed in Supplementary Table 1.

### Subcellular Localization of VviMYB86

The ORF of *VviMYB86* without the stop codon was PCR-amplified with specific primers (Supplementary Table 1) and subsequently inserted into a pEZS-NL expression vector containing the green fluorescent protein (GFP) reporter gene. Next, the specific procedure followed the method described previously (Meng et al., 2020). An empty vector was not used as a control because the “NL” vector could not express GFP well in the absence of a coding sequence added to the 5′ end of the ORF^6^.

### Transactivation Assay of VviMYB86 in Yeast

A transactivation assay of VviMYB86 in yeast (*Saccharomyces cerevisiae*) was performed following the method described by Wang et al. (2016). The ORFs of *VviMYB86*, *VviMYBPAR* (GenBank No. AB911341), and *VviMYBC2-L1* (GenBank No. EU181425) were cloned with specific primer pairs (Supplementary Table 1) and integrated into the yeast expression vector pGBKT7 (Takara, Japan). The expression vectors pGBKT7-VviMYB86, pGBKT7-VviMYBPAR (positive control), pGBKT7-VviMYBC2-L1 (negative control), and pGBKT7 (negative control) were transferred into a yeast host strain AH109 according to the manufacturer’s protocol (PT4087-1, Clontech, Japan). Successfully transformed yeast strains were applied onto the corresponding medium (SD/Trp-, SD/Trp-/His-, and SD/Trp-/His-/Ade-) and subsequently observed after being incubated at 30°C for 3–5 days.

### Transient Expression Using Arabidopsis Protoplasts and Dual Luciferase Assays

To test the transcription function of *VviMYB86*, dual luciferase assays were conducted using polyethylene glycol-mediated transformation of Arabidopsis protoplasts. The effector was generated by inserting the ORF region of *VviMYB86* into a pCAMBIA 1301 vector driven by the CaMV 35S promoter *via* an In-fusion HD cloning procedure (Takara, Japan). The empty vector was employed as the negative control. The promoter regions of *VviLAR1* (p*VviLAR1*), *VviLAR2* (p*VviLAR2*), *VviANR* (p*VviANR*), *VviANS* (p*VviANS*), *VviUFGT* (p*VviUFGT*), *VviMYBPA1* (p*VviMYBPA1*), *VviMYBPA2* (p*VviMYBPA2*), *VviMYBPAR* (p*VviMYBPAR*), *VviMYBC2-L1* (p*VviMYBC2-L1*), and *VviMYBA1* (p*VviMYBA1*) were amplified by PCR from the genomic DNA of CS by using gene-specific primers (Supplementary Table 1). Then, the promoter fragments were subcloned into a pGreenII 0800-LUC vector to serve as reporter plasmids. Protoplasts were extracted from young leaves of Arabidopsis, and then the effector and corresponding reporter genes were co-transformed into protoplasts as described previously (Mathur and Koncz, 1998). After being cultured in the dark at 23°C for 16 h, the transfected protoplasts were collected for dual luciferase assays using the Dual-Luciferase^®^ Reporter Assay System (Promega, United States) following the manufacturer’s protocol (VPE1910, Promega, United States). The relative luciferase activity was calculated as the ratio of firefly luciferase activity to Renilla luciferase activity. All the transfection experiments were performed in triplicate.

### Construction of the VviMYB86 Overexpression Vector and Grape Callus Transformation

The ORF of *VviMYB86* was inserted into a pCXSN vector under the control of the CaMV 35S promoter. Subsequently, the successfully constructed expression cassette was introduced into *A. tumefaciens* strain GV3101 by using the freeze-thaw method. Three-week-old wild-type (WT) callus was chosen for genetic transformation following the method described by Van Eck et al. (2006) and Meng et al. (2020). The subculture medium was solid B5 medium supplemented with 30 g/L sucrose, 2.5 g/L acid-hydrolyzed casein, 3.0 g/L phytagel, 0.1 mg/L α-naphthylacetic acid, 0.2 mg/L kinetin, and 5 mg/L hygromycin, and the pH was 5.9–6.0. The culture conditions and the subculturing period were the same as those applied to the WT callus. *VviMYB86* transgenic callus was confirmed by hygromycin-based gene detection and target gene expression quantification. The primers utilized in this section are listed in Supplementary Table 1.

### Light Treatment of WT Callus and VviMYB86 Transgenic Grape Callus

Subcultured WT and *VviMYB86* transgenic callus were employed to investigate the effects of light on PA and anthocyanin production, as well as the expression of *VviMYB86*, flavonoid-specific structural genes and related regulatory genes. For light treatment, the dark-cultured callus was subjected to a 16/8-h light/dark photoperiod with a light intensity of 2,000 lux for a subculturing period. The temperature was set at 24°C. After treatment, the callus was collected immediately for RNA extraction and production extraction. Three independent biological replicates were performed. The RNA extraction and qRT-PCR procedures were consistent with those described in section 2.3. *VviUbiquitin1* was selected as the reference gene, and the gene-specific primers used in this study are listed in Supplementary Table 1. The extraction and determination procedures for the PAs and anthocyanins are described in sections “4-Dimethylaminocinnamaldehyde (DMACA) Staining and PA Content Determination” and “Determination of the Total Anthocyanin Content”, respectively.

### 4-Dimethylaminocinnamaldehyde (DMACA) Staining and PA Content Determination

The presence of PAs in grape callus was detected by DMACA staining (Li et al., 1996). Fresh callus was stained with DMACA reagent [1% (w/v) in methanol: 6 M HCl (1:1, v/v)] for 1 h and then observed. Three biological replicates were performed for each set of samples. Approximately 1.0 g of frozen sample was ground into powder in liquid nitrogen for PA extraction. The specific procedure for the extraction of soluble and insoluble PAs followed the methods described by Yu et al. (2019). After extraction, soluble PAs were lyophilized and redissolved in 300 μL of 50% (v/v) methanol solution. The soluble PAs were quantified by the DMACA method. Soluble PA fractions (20 μL) were mixed with 100 μL of DMACA reagent on a 96-well plate. After samples were incubated at room temperature for 4 min, spectrophotometric quantification was performed at 640 nm using a SpectraMax 190 Microplate Reader (Molecular Devices, United States). (+)-Catechin (Sigma, United States) was used as a standard and processed in parallel with experimental samples. The insoluble PA content was analyzed by the butanol/HCl method. For quantification, one hundred microliters of supernatant were added to a 96-well plate, and then the A550 value was measured *via* a SpectraMax 190 Microplate Reader. Procyanidin B1 (Sigma, United States) was used as a standard.

### Determination of the Total Anthocyanin Content

Total anthocyanins were extracted with the HCl-methanol method (Liu et al., 2016), with slight modifications. Approximately 1.0 g of frozen sample was ground into powder in liquid nitrogen. Next, the powder was extracted with 1.0 mL of a hydrochloric acid: methanol solution (60% methanol; 0.1% hydrochloric acid) by sonicating in ice water for 30 min in the dark. The mixture was centrifuged at 8,000 rpm for 5 min at 4°C. The supernatant was collected and the residue was extracted twice. All the supernatants were pooled. The resulting supernatant was lyophilized and redissolved in 400 μL of hydrochloric acid: methanol solution. The extraction solution was finally stored at −80°C. The total anthocyanin content was determined by using the pH-differential method (Stojanovic and Silva, 2006). The extraction was diluted with KCl buffer (pH 1.0) and CH_3_CO_2_Na⋅3H_2_O buffer (pH 4.5). Next, spectrophotometric quantification of the mixture was performed at 520 and 700 nm using a SpectraMax 190 Microplate Reader. The total anthocyanin content was calculated using the following formula: total anthocyanin content = (A × MW × DF × V_e_ × 1,000)/(ε × *M*), where A = (A_520_ − A_700_)_pH1__.0_ − (A_520_ − A_700_)_pH4__.5_, MW is the molecular weight of malvidin-3-glucoside (493.5), DF is the dilution factor, V_e_ is the extraction volume, ε is the molar extinction coefficient of malvidin-3-glucoside (28,000), and *M* is the mass of the extracted samples.

### Statistical Analysis

The data are presented as the means ± standard deviations (SDs). One-way analysis of variance (ANOVA) was conducted using Microsoft Excel 2016 (Microsoft, United States). String diagrams and bar charts were created by GraphPad Prism 8 (GraphPad Software). Information regarding the experimental protocol, data analysis of RNA sequencing (RNA-seq) and submission to the NCBI Gene Expression Omnibus were recorded in the publication of our research group (Sun et al., 2019). In the present study, we selected the data of the control group at the E-L 29, E-L 31, and E-L 35 to E-L 38 stages of berry development for Pearson’s correlation analysis. Pearson’s correlation analysis was conducted using the fragments per kilobase of exon per million fragments value of each gene in R environmental language (version 3.5.1). TFs with correlation coefficients ≥0.7 or ≤0.7 and *P* < 0.05 were considered to have a significant expression correlation with the target gene.

## Results

### Isolation and Characterization of VviMYB86

To explore additional putative TFs involved in regulating PA biosynthesis, especially the expression of the genes encoding LAR in grape berries, we reanalyzed the RNA-seq database of the whole developmental period of CS berries deposited by Sun et al.^7^. An R2R3-MYB gene, annotated as *VviMYB86*, whose expression was strongly positively correlated with the expression of PA-specific structural and regulatory genes, including *VviLAR1*, *VviLAR2*, *VviANR*, and *VviMYBPAR*, was screened; the Pearson’s coefficients were 0.733, 0.923, 0.796, and 0.961, respectively (Supplementary Table 2). In addition, a co-expression analysis between *VviMYB86* and the genes involved in the anthocyanin biosynthesis branch was also performed. The Pearson’s coefficients between the expression levels of *VviMYB86* and *VviANS* and between those of *VviMYB86* and *VviUFGT* were −0.201 and −0.811, respectively (Supplementary Table 2). Therefore, we hypothesized that *VviMYB86* might participate in the regulation of both PA and anthocyanin biosynthesis in grape berries.

**FIGURE 1:**
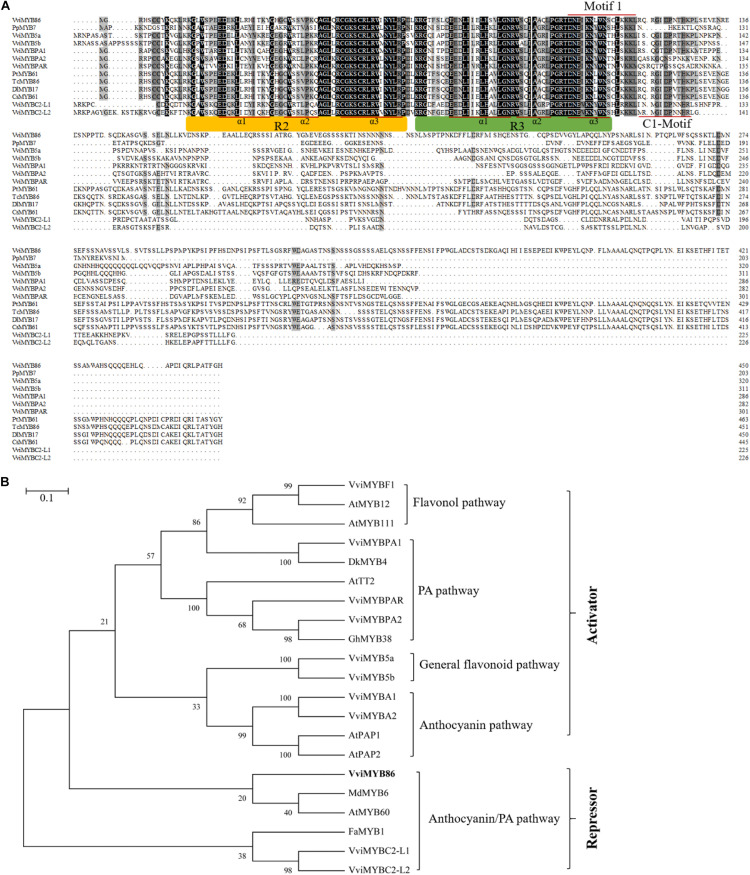
The deduced peptide sequences of VviMYB86 and related MYB’s. **(A)** Polypeptide alignment analysis of the full-length amino acid sequences of VviMYB86 and other PA-related R2R3 MYB transcription factors (TFs) as well as TFs with high homology. Conserved residues were highlighted in black, and partial conservation was indicated in gray. The R2 and R3 MYB DNA binding domains were indicated above the alignment. The alpha helices of R2 and R3 repeats were indicated with red lines. Motif 1 [DNEI(A/S/G)N(D/A/N)V] and C1-Motif (lsrGIDPxT/NHR) were indicated by underlining with red line. **(B)** Phylogenetic tree of MYB TFs related to VviMYB86. The phylogenetic tree was constructed using Neighbor-Joining method of MEGA 7.0 software. The scale bar represents the number of substitutions per site. The putative regulatory functions of most of the proteins in the control of flavonoid biosynthesis are indicated. GenBank accession numbers are as follows (in parentheses): AtTT2 (Q9FJA2), AtMYB12 (NP_182268), AtMYB111 (NP_199744), AtMYB60 (AAC83617), AtPAP1 (AAG42001), AtPAP2 (NP_176813), VviMYB5a (AAS68190), VviMYB5b (AAX51291), VviMYBPA1 (CAJ90831), VviMYBPA2 (ACK56131), VviMYBPAR (XP_003633091), VviMYBF1 (ACT88298), VviMYBA1 (BAD18977), VviMYBA2 (BAD18978), VviMYBC2-L1 (ABW34393), VviMYBC2-L2 (ACX50288), DkMYB4 (BAI49721), FaMYB1 (AAK84064), GhMYB38 (AAK19618), GhMYB10 (ABV53918), and MdMYB6 (AAZ20429).

Next, the 1,356-bp ORF of *VviMYB86* was amplified from the cDNA library of CS berries previously constructed by our lab (Mu et al., 2014; Supplementary Figure 2). Bioinformatic analysis indicated that *VviMYB86* encoded a predicted hydrophilic protein containing 451 amino acid residues and had a molecular mass of 50.23 kD and a pI of 6.47. Amino acid sequence analysis showed that, similar to other R2R3-MYB members, VviMYB86 processed two adjacent MYB repeats in the conserved DNA-binding domain, in which the R2 region was located between residues 14–61, and R3 was located between residues 67–111 (Figure 1A). A conserved C1 motif (lsrGIDPxT/NHR) (Chen et al., 2019) was found between residues117–128, which also existed in VviMYB5a, VviMYB5b, VviMYBPA1, VviMYBC2-L1, and VviMYBC2-L2. Moreover, VviMYB86 contained a conserved motif 1 [DNEI(A/S/G)N(D/A/N)V], which has been proven to bind to a specific site in bHLH proteins (Zimmermann et al., 2004; Wang et al., 2018), suggesting that VviMYB86 may interact with bHLH-type proteins. Phylogenetic analysis demonstrated that VviMYB86 was clustered with MdMYB6 and AtMYB60, and these TFs all had a negative effect on anthocyanin biosynthesis *in planta* (Figure 1B). The above results suggested that VviMYB86 was likely a candidate repressor of flavonoid biosynthesis.

### Subcellular Localization and Transcriptional Activity of VviMYB86

**FIGURE 2:**
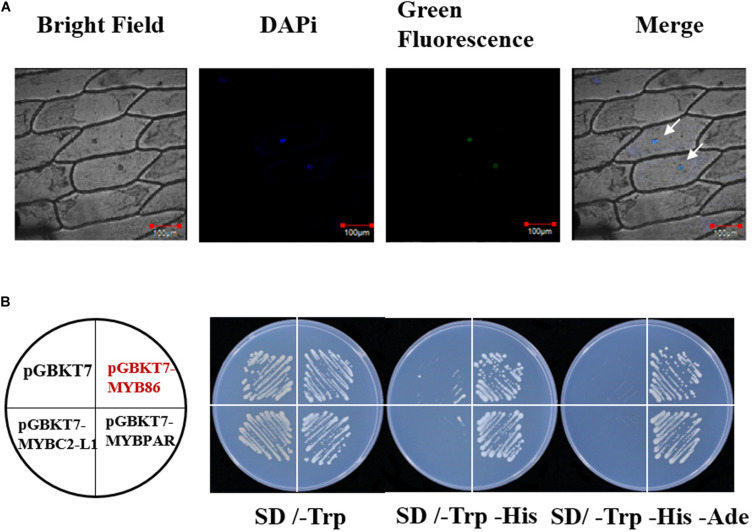
Subcellular localization of VviMYB86 and its transcription activation. **(A)** GFP activity in onion epidermal cells transiently expressing a *VviMYB86-GFP* construct. Bar: 100 μm. GFP, green fluorescence channel; Marker, blue fluorescence channel; DIC, bright light channel; Merge, the GFP and DAPi overlap. Arrows were the sites of GFP and DAPi overlap. **(B)** Transcription activation by VviMYB86 in yeast. The pGBKT7-VviMYBPAR plasmid was employed as a positive control. The pGBKT7-VviMYBC2L1 and pGBKT7 plasmids were employed as the negative controls. SD/-Trp, SD medium lacking tryptophan; SD/-Trp –His, SD medium lacking both tryptophan and histidine; SD/-Trp –His -Ade, SD medium lacking tryptophan, histidine, and adenine.

To examine the subcellular localization of the VviMYB86 protein, a *VviMYB86*-*GFP* expression cassette was generated and transformed into onion (*Allium cepa* L.) epidermal cells *via* particle bombardment. Confocal microscopy results revealed that cells expressing the *VviMYB86*-*GFP* fusion gene exhibited fluorescence restricted to the cell nucleus (Figure 2A), indicating that VviMYB86 was localized in the nucleus. In a yeast-based transactivation assay, like the positive control cells expressing GAL4 BD-VviMYBPAR, the yeast cells expressing the fusion protein GAL4 BD-VviMYB86 were able to grow on SD medium lacking leucine, histidine, and adenine, whereas the negative control yeast cells containing pGBKT7-VviMYBC2-L1 or the empty vector were able to grow only on SD medium lacking tryptophan (Figure 2B). These results indicated that VviMYB86 possessed transcriptional activity.

### Spatiotemporal Expression Patterns of VviMYB86 in Grapevines

**FIGURE 3:**
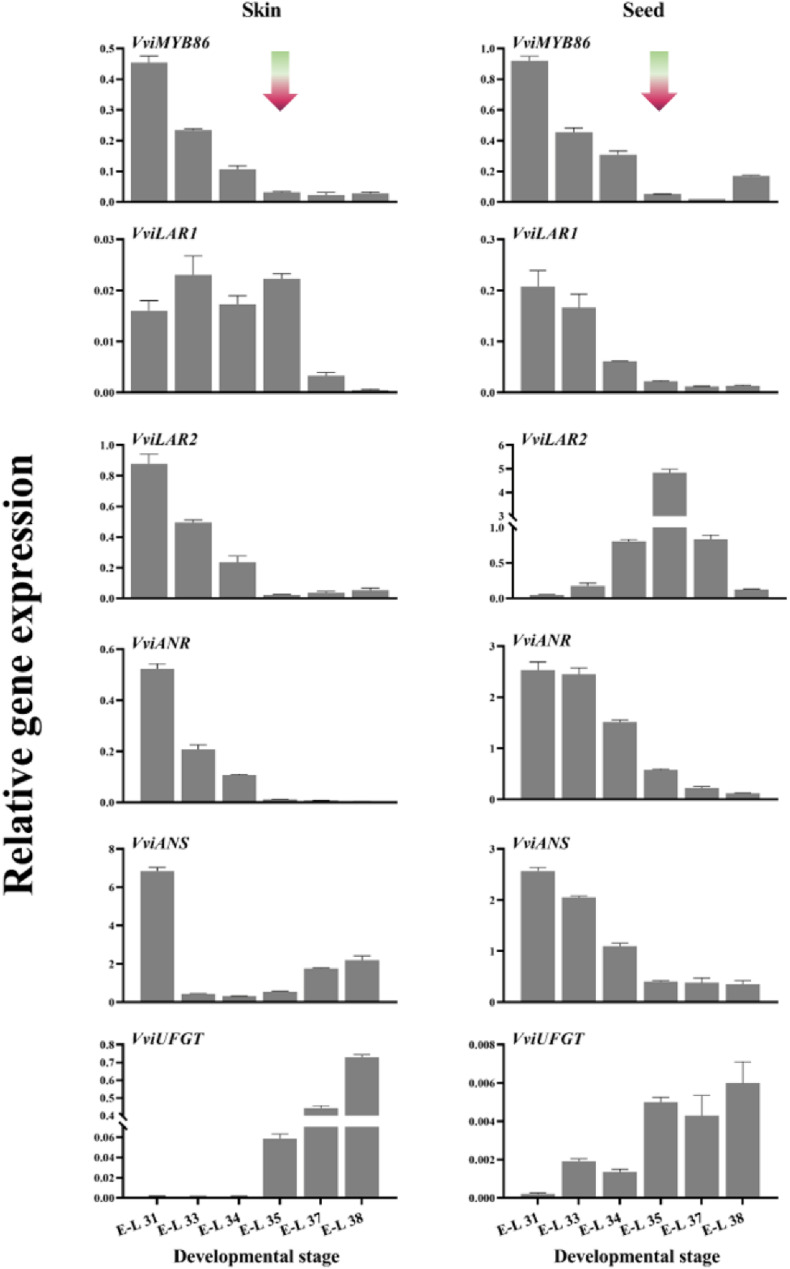
Spatiotemporal expression profile of *VviMYB86* and some PA-related and anthocyanin-related genes in grape skins and seeds. The gradient arrows indicated the time-point of véraison. Gene expression was determined by real-time quantitative PCR and normalized with the expression of *VviUbiquitin1*. Each sample was individually assayed in triplicate. Error bars indicated the standard error of the mean.

It has been found that grape flavonoids preferentially localize in both grape berry skins and seeds but are present in only negligible amounts in the mesocarp (Adams, 2006; Braidot et al., 2008). Therefore, the expression patterns of *VviMYB86* during grape berry development were assessed by qRT-PCR with RNA isolated from the skins and seeds of CS berries (Figure 3). In both berry skins and seeds, the expression level of *VviMYB86* was high in the early developmental stage, from E-L 31 to E-L 34 (Figure 3). However, the expression of this gene progressively decreased throughout the entire developmental stage of berry skins and seeds. The expression pattern of *VviMYB86* in whole berry development coincided well with the expression of PA-specific structural genes in grape berries, whereas it contrasted with that of *VviUFGT* in berry skins (Figure 3).

In addition, the transcript levels of *VviMYB86* in different grapevine organs, including roots, tendrils, flowers, stems, and young and mature leaves, were quantified by qRT-PCR. *VviMYB86* was expressed in all the examined grapevine organs with different transcript levels. Specifically, *VviMYB86* was most abundantly expressed in tendrils followed by flowers, stems, and then young and mature leaves, with a relatively low level of expression in the roots (Supplementary Figure 3).

The above results indicated that the expression of *VviMYB86* was regulated in a tissue- and temporal-specific manner in grapevine.

### Promoter Activation of PA and Anthocyanin Biosynthetic Pathway Genes in the Arabidopsis Protoplast System

**FIGURE 4:**
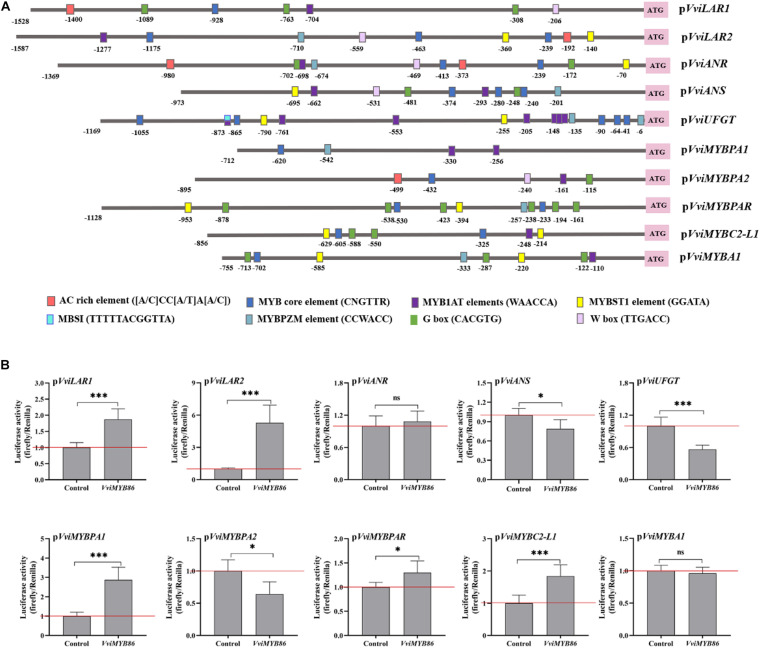
The influence of *VviMYB86* on the promoters of structural and regulatory genes in PA and anthocyanin synthesis. **(A)** The characteristics of *cis*-elements for binding of MYB, MYC and other proteins in structural and regulatory gene promoters of PA and anthocyanin synthesis. **(B)** Effects of *VviMYB86* on the activities of gene promoters of PA and anthocyanin synthesis as determined by dual luciferase assay. Control indicated the activity of the promoter transfected with the empty vector (pCAMBIA 1301). Normalized luciferase activity was calculated as the ratio between firefly and *Renilla reniformis* luciferase activities. Each column represents means ± SD from four biological replicates.

To identify the target PA and anthocyanin pathway genes of *VviMYB86* in grape berries, dual luciferase assays were performed using the Arabidopsis protoplast system. First, the promoters of the structural and regulatory genes in the PA and anthocyanin biosynthesis branches, including *VviLAR1*, *VviLAR2*, *VviANR*, *VviANS*, *VviUFGT*, *VviMYBPA1*, *VviMYBPA2*, *VviMYBPAR*, *VviMYBC2-L1*, and *VviMYBA1*, were cloned from the genomic DNA of grape berries; the lengths were 1,528, 1,587, 1,369, 973, 1,169, 712, 895, 1,128, 856, and 755 bp, respectively. Next, the *cis*-elements existing on these gene promoters were predicted by the PlantCARE database (see text footnote 5). As shown in Figure 4A, all ten gene promoters contained at least one of the following types of predicted MYB-binding element: the AC-rich element [(A/C)CC(A/T)A(A/C)], the MYB core element (CNGTTR), the MBSI (TTTTTACGGTTA), the MYBST1 element (GGATA), the MYB1AT element (WAACCA), and the MYBPZM element (CCWACC) (Xu et al., 2015; Zhou et al., 2019), indicating that all ten genes could be regulated by MYB TFs. In addition, the predicted bHLH-binding site G box (CACGTG) was observed in p*VviLAR1*, p*VviANR*, p*VviANS*, p*VviMYBPA2*, p*VviMYBPAR*, p*VviMYBC2-L1*, and p*VviMYBA1* (Hichri et al., 2011), which suggested that the seven genes could also be regulated by TFs from the bHLH family. Except for p*VviUFGT*, p*VviMYBPA2*, p*VviMYBPAR*, p*VviMYBC2-L1*, and p*VviMYBA1*, five other gene promoters contained a WRKY-binding site W box (TTGACC) (Shang et al., 2010), implying that WRKY family TFs might participate in regulating these genes, which still need to be proven experimentally.

Then, *VviMYB86* and the different aforementioned gene promoters were co-transformed into Arabidopsis protoplasts, and dual luciferase assays were conducted (Figure 4B). The results demonstrated that *VviMYB86* strongly enhanced the activities of p*VviLAR1* (by approximately 1.87-fold) and p*VviLAR2* (by approximately 5.29-fold) but not p*VviANR* (by approximately 1.08-fold). In contrast, *VviMYB86* repressed the activities of p*VviANS* and p*VviUFGT*, causing 21% and 44% decreases in the activity of p*VviANS* and p*VviUFGT*, respectively, compared with that of the control. Furthermore, *VviMYB86* could also have an effect on the activity of the promoters of several regulatory genes related to PA and anthocyanin biosynthesis. In detail, *VviMYB86* enhanced the activities of p*VviMYBPA1* (by approximately 2.88-fold) and p*VviMYBPAR* (by approximately 1.30-fold), whereas repressed the activity of p*VviMYBA1* but not significantly.

In summary, *VviMYB86* might regulate the expression of structural genes encoding LAR and consequently regulate PA biosynthesis in grape berries, and the VviMYB86 signaling might act upstream of VviMYBPA1 and VviMYBPAR. In terms of anthocyanin synthesis, *VviMYB86* might exert a negative effect by downregulating the expression of *VviANS* and *VviUFGT*.

### Promotion of PA Biosynthesis in Grape Callus by the Overexpression of VviMYB86

**FIGURE 5:**
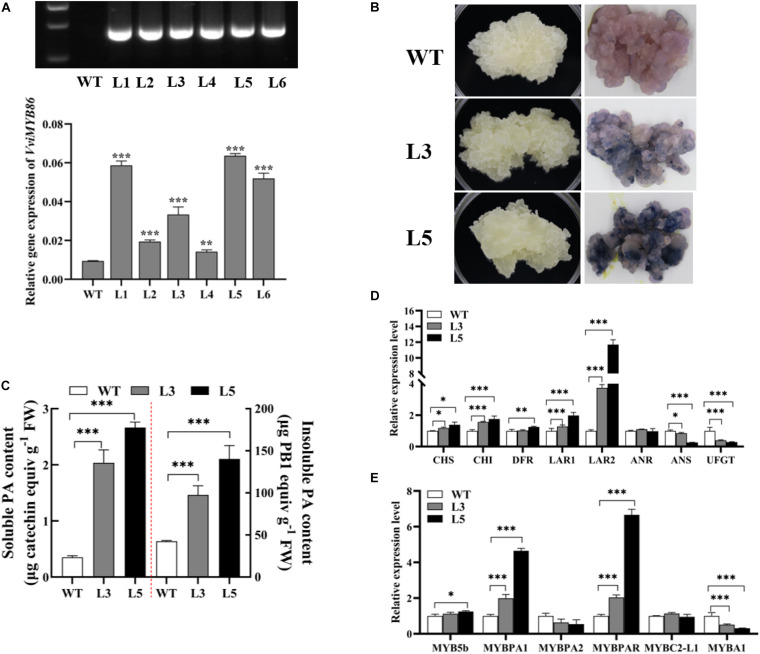
*VviMYB86* positively regulated proanthocyanidin (PA) synthesis in grape callus. **(A)** Identification of transgenic callus. The top was hygromycin gene expression detected by PCR and analyzed by gel electrophoresis. The below was the expression level of *VviMYB86* in wild type (WT) callus and different transgenic lines (L1-L6). The value above the column referred to the transcripts of *VviMYB86* in transgenic and WT calluses cultured under dark conditions. **(B)** The photos of WT callus and two independent *VviMYB86* transgenic lines (L3 and L5). The left was WT callus and transgenic lines in their natural growth states cultured in darkness. The right was WT callus and transgenic lines after 4-Dimethylaminocinnamaldehyde (DMACA) staining. **(C)** The soluble and insoluble PA content in the WT callus and transgenic lines. FW, fresh weight. Data was expressed as means ± SD of three replicates. Asterisks indicated significant differences relative to the control by one-way ANOVA test (^∗∗∗^*p* < 0.001). **(D)** The relative expression of flavonoid pathway related structural genes. After several successive rounds of subculture, stable transgenic callus lines were established on selectable medium. Callus grown for 25 days was collected for each assay. CHS, chalcone synthase; CHI, chalcone isomerase; DFR, dihydroflavonol-4-reductase; LAR, leucoanthocyanidin reductase; ANR, anthocyanidin reductase; ANS, anthocyanidin synthase; UFGT; UDP-glucose: flavonoid-3-*O*-glucosyltransferase. **(E)** The relative expression of known flavonoid regulators. Data was expressed as means ± SD of three replicates. Asterisks indicated significant differences relative to the control by one-way ANOVA test (^∗^*p* < 0.05; ^∗∗^*p* < 0.01; ^∗∗∗^*p* < 0.001).

To demonstrate the function of *VviMYB86* on PA biosynthesis in grape berries, we generated *VviMYB86*-overexpressing transgenic grape callus. WT callus was used as the control. The culture conditions for both the WT and transgenic calluses were 24 h of darkness at a temperature of 24°C. The relative expression levels of *VviMYB86* in the WT callus and *VviMYB86* transgenic lines were determined by qRT-PCR. Three independent transgenic lines (L1, L3, and L5) with different ectopic expression levels of *VviMYB86* (Figure 5A and Supplementary Figure 4) were selected for further analysis. The morphological features of the transgenic lines were similar to those of the WT callus, and both were white and loose (Figure 5B and Supplementary Figure 5A). After being stained with DMACA for 1 h, all the transgenic lines exhibited a notable dark-blue color, whereas the color of the WT callus was pink, suggesting that the *VviMYB86* transgenic lines accumulated more PAs than the WT callus (Figure 5B and Supplementary Figure 5A). The determination of both PA and insoluble contents through the DMACA and butanol/HCl methods, respectively, also confirmed that, compared with the WT callus, the three *VviMYB86* transgenic lines produced large amounts of both soluble and insoluble PAs (Figure 5C and Supplementary Figure 5B).

To confirm the target genes of *VviMYB86*, the expression levels of genes participating in the flavonoid biosynthesis pathway were determined by qRT-PCR. The results demonstrated that the expression of most genes related to the PA biosynthesis pathway was upregulated in the *VviMYB86* transgenic calluses (Figure 5D and Supplementary Figure 5C). Moreover, in the *VviMYB86* transgenic calluses, the expression of all the upstream genes including *VviCHS*, *VviCHI*, *VviFLS*, and *VviDFR*, was all upregulated to different degrees. With respect to PA-specific genes, the expression of *VviLAR1* and *VviLAR2* was enhanced, especially *VviLAR2*, whose expression was upregulated 3.16-fold in L1, 2.73-fold in L3 and 10.68-fold in L5. However, in all the transgenic lines, the expression level of *VviANR* remained unchanged compared with that in the WT callus (Figure 5D and Supplementary Figure 5C). These results suggested that *VviMYB86* tended to primarily modulate the expression of the two LAR genes, subsequently leading to increased PA contents. Furthermore, the expression levels of known PA regulators, including *VviMYB5a*, *VviMYB5b*, *VviMYBPA1*, and *VviMYBPAR*, increased to different levels in the *VviMYB86* transgenic calluses relative to those in the WT callus (Figure 5E and Supplementary Figure 5D).

### VviMYB86 Represses the Anthocyanin Biosynthesis Branch in Transgenic Grape Callus Under Light Conditions

We observed that the expression levels of anthocyanin pathway genes, including *VviANS* and *VviUFGT*, were repressed in the *VviMYB86* transgenic lines, compared with those in the WT callus (Figure 5D and Supplementary Figure 5C). However, anthocyanins were detected at very low levels in both WT callus and *VviMYB86* transgenic lines cultured under dark conditions (Supplementary Figure 6). However, the effect of overexpressing *VviMYB86* on anthocyanin accumulation has not been fully determined.

**FIGURE 6:**
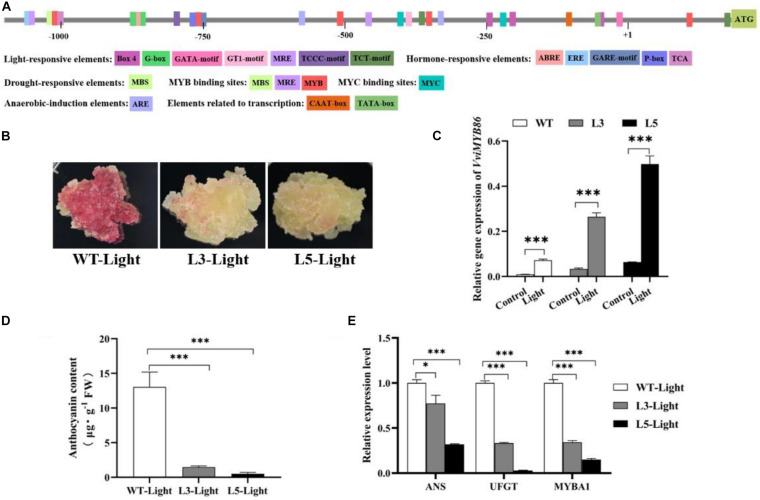
*VviMYB86* negatively regulated anthocyanin synthesis in grape callus. WT-Light, wild type callus cultured under light conditions; L3-Light, transgenic line 3 cultured under light conditions; L5-Light, transgenic line 5 cultured under light conditions. **(A)** The *cis*-acting elements present in *VviMYB86* promoter predicted by PlantCARE website (http://bioinformatics.psb.ugent.be/webtools/plantcare/html/). **(B)** The nature status of WT callus and transgenic line 3 and line 5 under light conditions. **(C)** The expression levels of *VviMYB86* in WT and transgenic calluses. Control: wild-type callus and transgenic calluses that cultured under dark conditions. Light: wild-type callus and transgenic calluses that cultured under light conditions. The value above the column referred to the transcripts of *VviMYB86* in transgenic and WT calluses. **(D)** Anthocyanin contents in the WT callus and transgenic lines cultured under light conditions. The anthocyanin contents in callus samples measured by using the pH-differential method. FW, fresh weight. **(E)** Expressions of corresponding genes of anthocyanin biosynthesis pathway in the WT callus and transgenic lines cultured under light conditions. ANS, anthocyanidin synthase; UFGT, UDP-glucose: flavonoid-3-*O*-glucosyltransferase. Data was expressed as means ± SD of three replicates. Asterisks indicated significant differences relative to the control by one-way ANOVA test (^∗^*p* < 0.05; ^∗∗∗^*p* < 0.001).

Then, the promoter region of *VviMYB86* was isolated from the genomic DNA of CS berries (Supplementary Figure 7). To elucidate the regulatory mechanisms that might control the expression of *VviMYB86*, p*VviMYB86* was subsequently analyzed by predicting the *cis*-elements though the PlantCARE database (see text footnote 5), and the results were shown in Figure 6A. Several *cis-*elements related to light response, environmental response, hormonal response and developmental regulation were found to be widely present in p*VviMYB86*, indicating that *VviMYB86* participated in multiple physiological processes in grape berries. Among these *cis*-elements, light-responsive elements were the most frequent and most variable, suggesting that the expression of *VviMYB86* may be regulated by light.

Next, the three independent *VviMYB86* transgenic lines cultured under dark conditions were subjected to light conditions (L1-Light, L3-Light, and L5-Light) with a light intensity of approximately 2,000 lux for a subculture period. WT callus subjected to the same light condition (WT-Light) was employed as the control. We observed that the WT-Light turned red, whereas the three transgenic lines were only slightly reddish in color (Figure 6B and Supplementary Figure 8A). The addition of light increased the transcription level of *VviMYB86* in the light-exposed transgenic lines as well as in the WT-Light compared with that in the corresponding calluses cultured under normal conditions (Figure 6C and Supplementary Figure 8B). The results revealed that the expression of *VviMYB86* could be inducible by light.

In addition, the transcript levels of *VviMYB86* in light-exposed transgenic lines were greater than those in the WT-Light (Figure 6C and Supplementary Figure 8B). Anthocyanins were subsequently extracted from both the light-exposed WT and *VviMYB86* transgenic calluses. The measurement of anthocyanin contents revealed that anthocyanin production was considerably reduced in the light-exposed transgenic calluses (Figure 6D and Supplementary Figure 8C). The qPCR results showed that the expression of anthocyanin biosynthesis branch genes, including *VviANS* and *VviUFGT*, was downregulated in the light-exposed transgenic lines (Figure 6E and Supplementary Figure 8D). In addition, VviMYBA1, a MYB TF, specifically regulates anthocyanin synthesis (Cutanda-Perez et al., 2009); the expression of *VviMYBA1* was also repressed (Figure 6E and Supplementary Figure 8D). Taken together, the above results suggested that *VviMYB86* repressed anthocyanin biosynthesis in grape callus cultured under light conditions.

## Discussion

### VviMYB86 Regulates PA Biosynthesis in Grape Berries Primarily by Affecting the Expression of the Two LAR Genes

In this study, we demonstrated that VviMYB86 may participate in the regulation of PA biosynthesis in grape berries. Unlike Arabidopsis, in which PAs are solely detected as monomeric or polymeric (−)-epicatechin forms, PAs are present as both (+)-catechin- and (−)-epicatechin-based PAs in grape skins and seeds (Xie et al., 2003; Bogs et al., 2005). The reason for this discrepancy is that LAR is not encoded in the Arabidopsis genome (Xie et al., 2003). In grapes, there are two LARs (VviLAR1 and VviLAR2) whose function are similar for PA biosynthesis (Yu et al., 2019). The temporal- and tissue-specific expression patterns of genes encoding LAR and *VviANR* contribute to the continuous accumulation of PAs before véraison in grape berries (Bogs et al., 2005). However, in grape berries, PA-related MYB TFs, including VviMYB5b, VviMYBPA1, VviMYBPA2, and VviMYBC2-L1, significantly influence the expression levels of *VviLAR1* and *VviANR* but not *VviLAR2* (Bogs et al., 2007; Deluc et al., 2008; Terrier et al., 2009; Huang et al., 2014). Although VviMYB5a activates the activity of only p*VviLAR1*, ectopic expression of *VviMYB5a* also activates the transcription of *AtBAN* (which encodes ANR) in Arabidopsis and induces the accumulation of (−)-epicatechin-form PAs but not (+)-catechin-form PAs in tobacco (Deluc et al., 2006, 2008). VviMYBPAR, whose gene transcription profile is closely related to that of *VviLAR2*, significantly activates the activities of p*VviLAR1* and p*VviANR*, whereas it only slightly increases the activity of p*VviLAR2* in a transient reporter assay (Koyama et al., 2014). Recently, by calculating the correlations between the expression levels of structural and regulatory genes in the PA biosynthesis branch of grapevine calluses subjected to different light intensities, researchers showed that *VviLAR2* was the target gene of VviMYBPA2. However, owing to the low expression level of *VviMYBPA2*, the effects on the expression level of *VviLAR2* are limited (Cheng et al., 2020).

Unlike known PA regulators, *VviMYB86* appeared to utilize *VviLAR1* and *VviLAR2* as target genes to control PA synthesis in grape berries. The spatiotemporal expression profiles of *VviMYB86* in grape berry skins and seeds were consistent with those of *VviLAR2* and *VviLAR1*, respectively. The results of our dual luciferase assay indicated that *VviMYB86* enhanced the activities of p*VviLAR1* and p*VviLAR2*. Overexpression of *VviMYB86* in grape callus that cultured under dark condition also enhanced the transcript levels of *VviLAR1* and *VviLAR2*. Our results may help to elucidate the regulatory mechanism governing the expression of the two LAR genes in grape berries.

In peach, PpMYB7 regulates PA synthesis by activating the transcription of *PpLAR1* but not *PpANR* (Zhou et al., 2015). With respect to members of the R2R3-MYB TF family, the α-helix regions of the R2R3 domain have been shown to play a critical role in directing MYB TFs to bind the promoters of flavonoid structural genes (Williams and Grotewold, 1997; Jia et al., 2004). The first and second α-helices of the R2 domain in both VviMYB86 and PpMYB7 differ from those of the other PA-related R2R3-MYB regulators, whereas the third α-helix of the R2 domain and all the three α-helices of the R3 domain are conserved. Jia et al. (2004) reported that the first two flexible α-helix regions of the R2 domain might be important for the recognition of a specific DNA-binding site, as they afford secondary structure and ternary folding of the R2R3 domain. Therefore, further experiments should be performed to address whether the difference in activation patterns between VviMYB86 as well as PpMYB7 and other PA-related R2R3-MYB regulators is caused by genetic variation in the region encoding the first and second α-helices of the R2 domain. Overall, both VviMYB86 and PpMYB7 represent a new group of R2R3-MYB genes that regulate PA biosynthesis in plants.

Furthermore, *VviMYB86* not only controls the expression of PA-specific structural genes in grape berries but also affects the expression of known PA regulators, especially *VviMYBPAR* and *VviMYBPA1*. The results of the dual luciferase assay revealed that *VviMYB86* enhanced the activities of p*VviMYBPA1* and p*VviMYBPAR*. Overexpression of *VviMYB86* in grape callus increased the transcript levels of *VviMYBPA1* and *VviMYBPAR*. It is possible that VviMYB86 signaling acts upstream of VviMYBPA1 and VviMYBPAR. Similar interactions have been suggested between VviMYBPA1 and VviMYBPA2, as well as VviMYBPAR and VviMYBPA1 (Terrier et al., 2009; Koyama et al., 2014).

### VviMYB86 Responds to Light and Represses Anthocyanin Biosynthesis

Light is an important environmental factor that influences flavonoid biosynthesis in plants. MYB TFs play pivotal roles in the response to external stimuli and in the regulation of flavonoid synthesis. The majority of MYB TFs associated with flavonoid synthesis have been found to respond to light, such as VviMYBPA1 (Koyama et al., 2012), VviMYBF1 (Azuma et al., 2012), and PyMYB10 (Zhang et al., 2011). The transcript levels of these R2R3-MYB TFs are modulated to regulate the biosynthesis of flavonoid compounds with changing light conditions (Zoratti et al., 2014). In this study, VviMYB86 was also confirmed to be affected by light, since its transcript levels were enhanced when *VviMYB86* transgenic callus and the WT callus were subjected to light conditions.

However, anthocyanin production and the expression of corresponding genes were repressed in the light-exposed *VviMYB86* transgenic lines compared with those in the WT-Light callus. These results demonstrated that *VviMYB86* obviously repressed anthocyanin synthesis under light conditions in grapes. The presence of both positive and negative regulators of anthocyanin-related structural genes keeps the anthocyanin levels *in planta* in balance (Ricardo Perez-Diaz et al., 2016). The expression pattern of *VviMYB86* is opposite that of anthocyanin biosynthesis, as well as that of *VviMYBA1*, throughout the entire developmental stages of grape berries (Cutanda-Perez et al., 2009). These negative correlations in developmental expression have also been observed with *VviMYBC2-L2* and *VviMYB4*-like (Ricardo Perez-Diaz et al., 2016; Zhu et al., 2019), suggesting that these MYB TFs are expressed in grapevine organs to inhibit ectopic anthocyanin accumulation in grape berries.

### VviMYB86 Oppositely Regulates PA and Anthocyanin Biosynthesis in Grapes

Several R2R3-MYB TFs have been verified to have a broad effect on the regulatory function of flavonoid biosynthesis in grape berries (Wei et al., 2020). In contrast to these functionally specialized types of R2R3-MYB TFs, such as VviMYBPA1 (Bogs et al., 2007), VviMYBPA2 (Terrier et al., 2009), and VviMYBPAR (Koyama et al., 2014), VviMYB86 modulates both PA and anthocyanin biosynthesis in grape berries. Unlike VviMYB5a (Deluc et al., 2006) and VviMYB5b (Deluc et al., 2008), both of which positively regulate PA and anthocyanin biosynthesis in grape berries, VviMYB86 had different regulatory mechanisms for PAs and anthocyanins. *VviMYB86* positively regulated the PA biosynthesis branch but negatively affected anthocyanin biosynthesis in grape berries. Recently, MYB TFs with different regulatory mechanisms for different biosynthesis branches of flavonoids have also been found in other plant species. In the Chinese narcissus (*Narcissus tazetta* L. var. *chinensis*), NtMYB3 induces PA accumulation by repressing flavonol biosynthesis (Anwar et al., 2019). In *Medicago truncatula*, MtPAR promotes PA accumulation by directly repressing isoflavone biosynthesis and by redirecting the flux of the immediate precursors into the PA biosynthesis branch (Li et al., 2016). In *MtMYB14*-overexpressing hairy roots, PA accumulation is strongly induced and anthocyanin production is decreased by half (Liu et al., 2014). *CsMYB5a*-overexpressing tobacco plants exhibit downregulated anthocyanin accumulation but present a high polymeric PA content in the flowers (Jiang et al., 2018). Hence, the results of our study help to elucidate the role and mechanism of VviMYB86 in the regulatory network governing the biosynthesis of anthocyanins and PAs in grape berries.

In summary, the results of this study show that *VviMYB86* is a candidate gene implicated in the regulation of different branches of the flavonoid biosynthesis pathway in grape berries. Our results prove that *VviMYB86* controls PA biosynthesis, primarily by affecting the expression of the two LAR genes in the Arabidopsis transient transformation system, as well as in grape callus, when overexpressed. Moreover, *VviMYB86* is observed to repress anthocyanin biosynthesis branch in grapes by downregulating the transcript levels of *VviANS* and *VviUFGT*. The results of this study help to elucidate the regulatory mechanism governing the expression of the two LAR genes in grape berries and provide new insights into the role and mechanism of VviMYB86 in the regulation of the metabolic flux between the PA and anthocyanin biosynthesis branches in grape berries.

## Data Availability Statement

The datasets presented in this study can be found in online repositories. The names of the repository/repositories and accession number(s) can be found in the article/Supplementary Material.

## Author Contributions

CD and JW conceived and guided the experiments. JC performed the research, analyzed the data, and wrote the original manuscript. KY and YS perfected the research scheme. All authors critically revised the article.

## Conflict of Interest

The authors declare that the research was conducted in the absence of any commercial or financial relationships that could be construed as a potential conflict of interest.

## Footnotes

1 http://genomes.cribi.unipd.it/grape/

2 http://www.ebi.ac.uk/Tools/pfa/iprscan/

3 http://web.expasy.org/protparam/

4 http://genomes.cribi.unipd.it/grape/

5 http://bioinformatics.psb.ugent.be/webtools/plantcare/html/

6 https://deepgreen.dpb.carnegiescience.edu/

7 https://www.ncbi.nlm.nih.gov/geo/query/acc.cgi?acc=GSE129916
