# Supplementary material for: Transcription Factor VviMYB86 Oppositely Regulates Proanthocyanidin and Anthocyanin Biosynthesis in Grape Berries
Source: Front Plant Sci. 2021 Jan 13;11:613677. doi: 10.3389/fpls.2020.613677 (PMC7838568; doi:10.3389/fpls.2020.613677)
Supplement: Supplementary file 1 [file Data_Sheet_1.docx]

Supplementary Materials


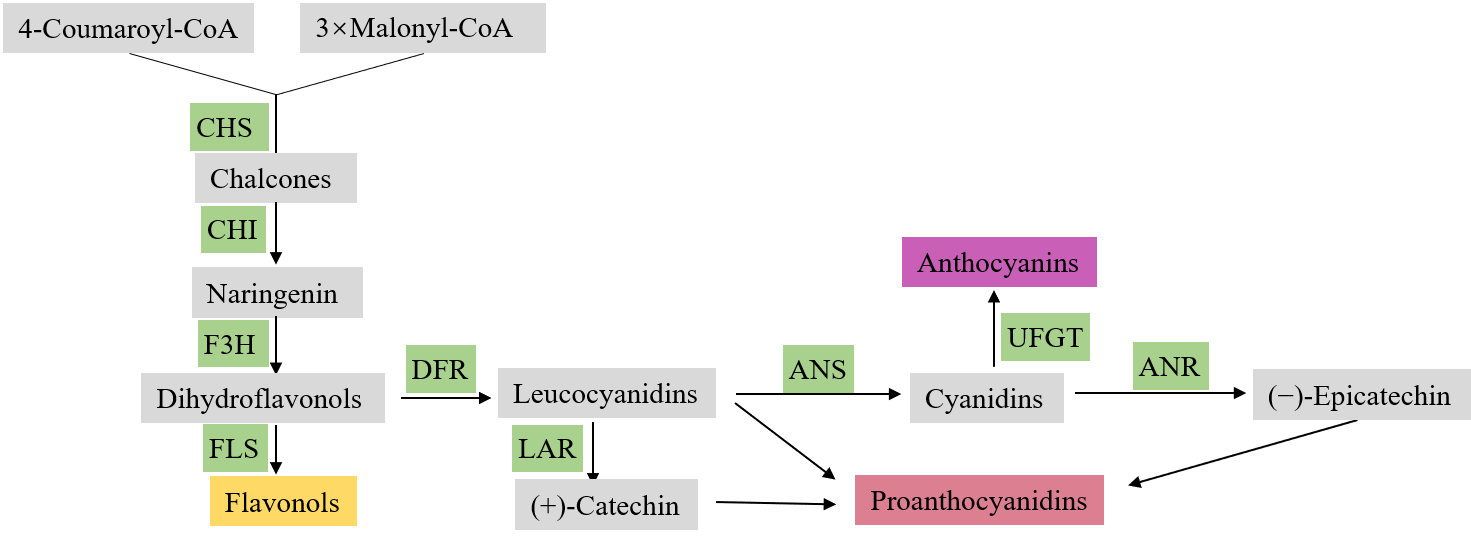


**Supplementary Figure 1** Simplified representation of flavonoid biosynthetic pathway leading to the production of proanthocyanidins (PAs) and anthocyanins of grapes. The enzyme names are abbreviated as follows: CHS, chalcone synthase; CHI, chalcone isomerase; F3H, flavonoid 3-hydroxylase; FLS, flavonol synthase; DFR, dihydroflavonol-4-reductase; ANS, anthocyanidin synthase; LAR, leucoanthocyanidin reductase; ANR, anthocyanidin reductase; UFGT, UDP-glucose: flavonoid-3-*O*-glucosyltransferase.


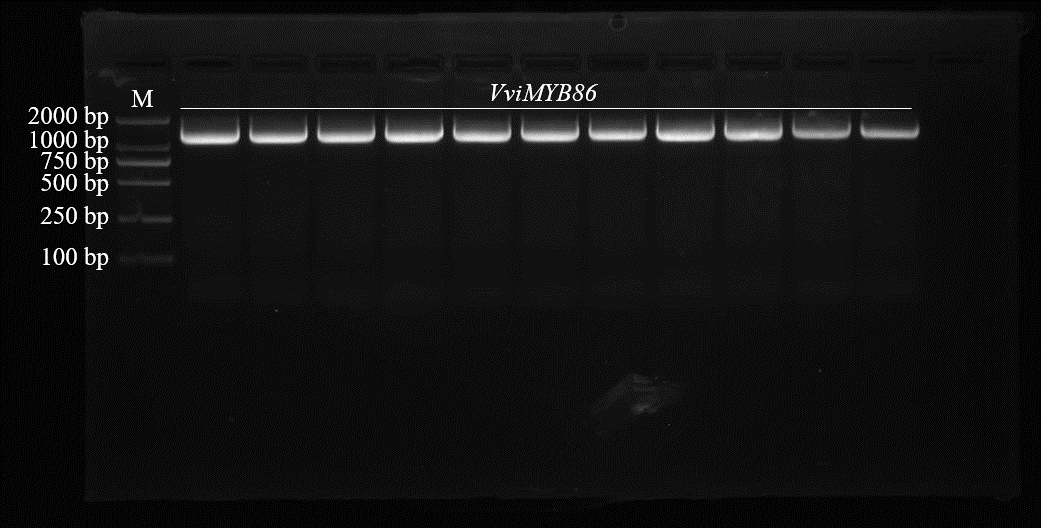


**Supplementary Figure 2** The origin gel of PCR products of the *VviMYB86* amplified from cDNA library of *Vitis vinifera* L. cv. Cabernet Sauvignon. M: DNA marker DL 2000 (Tiangen, China).


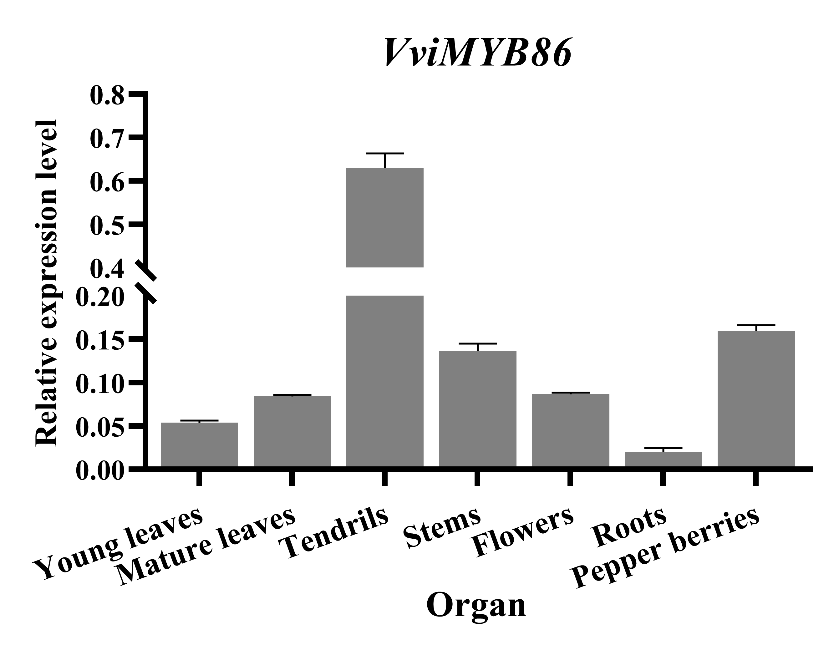


**Supplementary Figure 3** The expression patterns of *VviMYB86* in different grapevine organs. *VviUbiquitin1* was used as the reference gene.


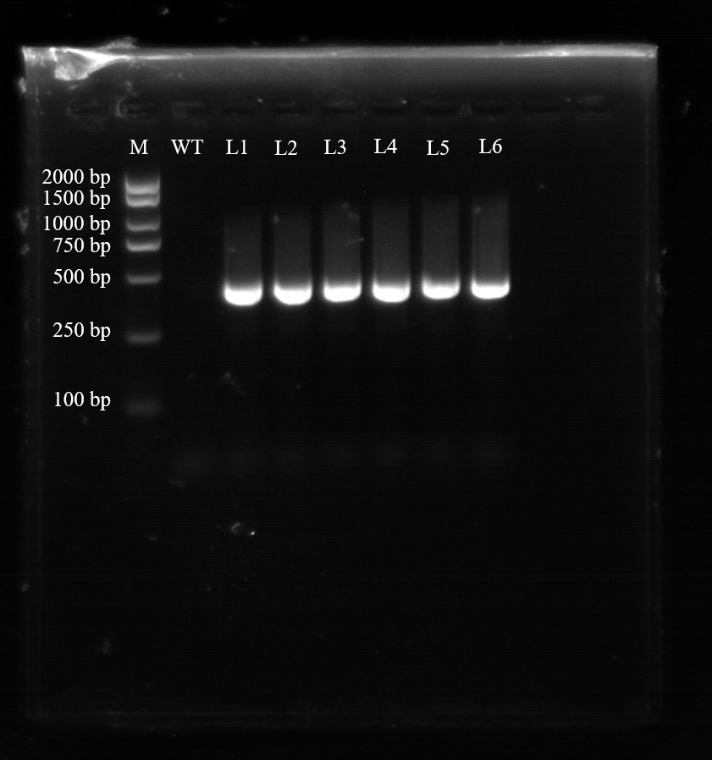


**Supplementary Figure 4** The origin gel of hygromycin gene expression detection in **Figure 5A**. M: DNA marker BM2000+ (Biomed Inc., China). WT, wild type grape callus. L1-L6, the six independent *VviMYB86* transgenic lines.


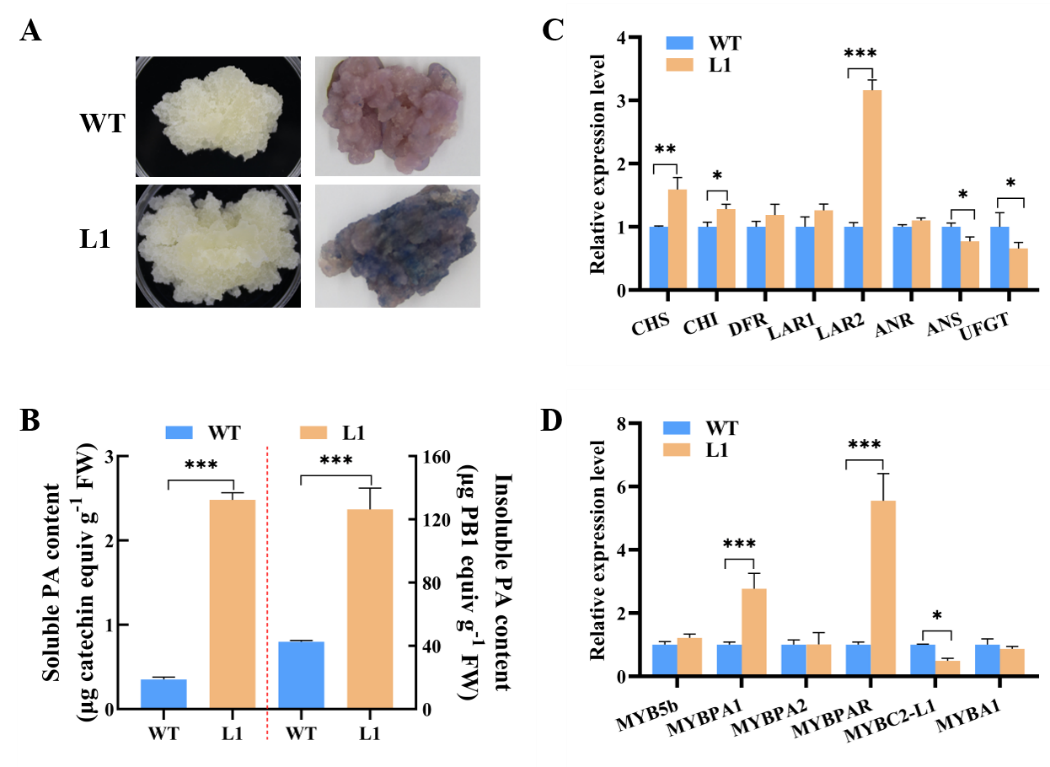


**Supplementary Figure 5** *VviMYB86* positively regulated proanthocyanidin (PA) synthesis in grape callus. (**A**) The photos of Wild-type (WT) callus and *VviMYB86* transgenic line 1 (L1). The left was WT callus and L1 in their natural growth states cultured in darkness. The right was WT callus and L1 after 4-Dimethylaminocinnamaldehyde (DMACA) staining. (**B**) The soluble and insoluble PA content in the WT callus and L1. FW, fresh weight. Data was expressed as means ± SD of three replicates. Asterisks indicated significant differences relative to the control by one-way ANOVA test (***, *p* < 0.001). (**C**) The relative expression of flavonoid pathway related structural genes. After several successive rounds of subculture, stable transgenic callus lines were established on selectable medium. Callus grown for 25 days was collected for each assay. CHS, chalcone synthase; CHI, chalcone isomerase; DFR, dihydroflavonol-4-reductase; LAR, leucoanthocyanidin reductase; ANR, anthocyanidin reductase; ANS, anthocyanidin synthase; UFGT, UDP-glucose: flavonoid-3-*O*-glucosyltransferase. (**D**) The relative expression of known flavonoid regulators. Data was expressed as means ± SD of three replicates. Asterisks indicated significant differences relative to the control by one-way ANOVA test (*, *p* < 0.05; ***, *p* < 0.001)

**
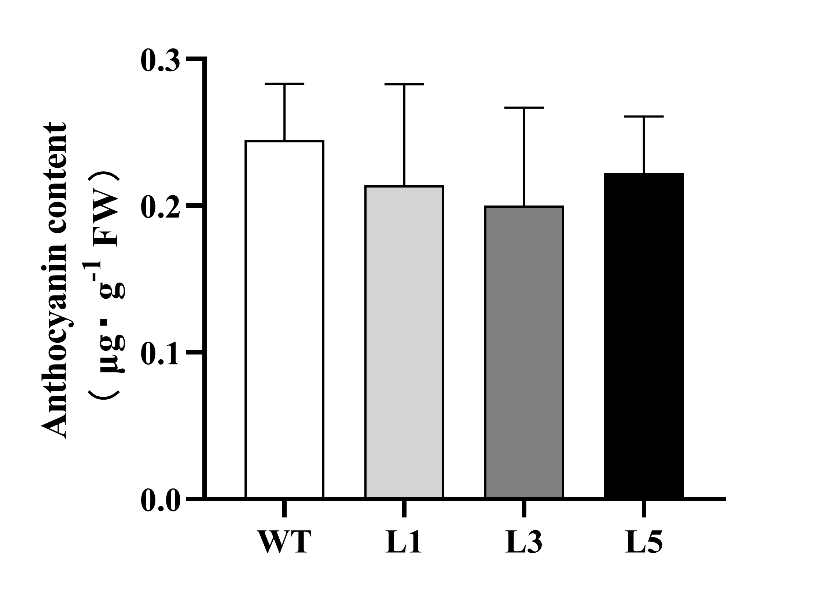
**

**Supplementary Figure 6** The anthocyanin content in the wild type callus (WT callus) and *VviMYB86* transgenic lines (L1, L3 and L5) cultured under dark conditions. FW, fresh weight. Data was expressed as means ± SD of three replicates.


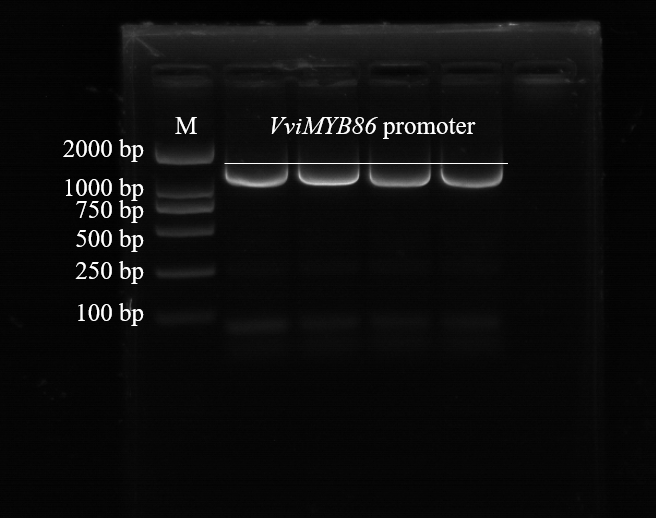


**Supplementary Figure 7** The origin gel of PCR products of the *VviMYB86* promoter amplified from genome DNA of *Vitis vinifera* L. cv. Cabernet Sauvignon. M: DNA marker DL 2000 (Tiangen, China).


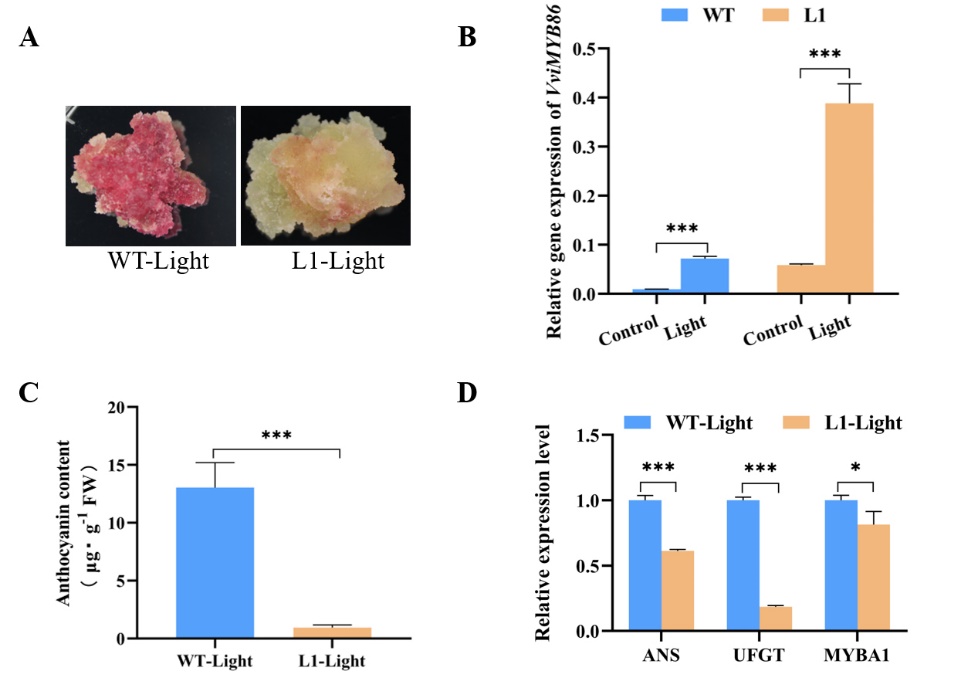


**Supplementary Figure 8** *VviMYB86* negatively regulated anthocyanin synthesis in grape callus. WT-Light, wild-type callus cultured under light conditions, L1-Light, transgenic line 1 cultured under light conditions. (**A**) The nature status of WT callus and transgenic line 1 under light conditions. (**B**) The expression levels of *VviMYB86* in WT and transgenic calluses. Control: wild-type callus and transgenic calluses that cultured under dark conditions. Light: wild-type callus and transgenic calluses that cultured under light conditions. The value above the column referred to the transcripts of *VviMYB86* in transgenic and WT calluses. (**C**) Anthocyanin contents in WT callus and transgenic line 1 cultured under light conditions. The anthocyanin contents in callus samples measured by using the pH-differential method. FW, fresh weight. (**D**) Expressions of corresponding genes of anthocyanin biosynthesis pathway in WT callus and transgenic line 1 cultured under light conditions. ANS, anthocyanidin synthase; UFGT, UDP-glucose: flavonoid-3-*O*-glucosyltransferase. Data was expressed as means ± SD of three replicates. Asterisks indicated significant differences relative to the control by one-way ANOVA test (*, *p* < 0.05, ***, *p* < 0.001).
